# A Case of Hypokalemic Periodic Paralysis in a Young Athlete

**DOI:** 10.7759/cureus.15236

**Published:** 2021-05-25

**Authors:** Randa Abdelmasih, Ramy Abdelmaseih, Mustajab Hasan, Hesham Nasser

**Affiliations:** 1 Internal Medicine, University of Central Florida College of Medicine, Orlando, USA

**Keywords:** hypokalemic periodic paralysis, acute periodic paralyses disorders, hypokalemia, paralysis, weakness, potassium, westphal's diease

## Abstract

Hypokalemic periodic paralysis (HPP) is one of the group muscle disorders that can cause sudden onset paresis or paralysis. It is a quite rare, yet, potentially life-threatening condition that, if appropriately and promptly diagnosed and treated, can be completely reversed. Other forms of periodic paralysis include thyrotoxic periodic paralysis, hyperkalemic periodic paralysis, and Anderson syndrome. We are presenting a case of a young male who presented to the emergency department (ED) with sudden paralysis to shed light on such a diagnosis and on other differential diagnoses.

## Introduction

Hypokalemic periodic paralysis (HPP), formally known as Westphal's disease, is an autosomal dominant channelopathy characterized by sudden muscle paresis or paralysis [1]. It is a quite rare, yet, potentially life-threatening condition that, if appropriately and promptly diagnosed and treated, can be completely reversed [2]. Here we present a rare case of HPP after a vigorous exercise activity to increase the awareness of such a differential diagnosis for sudden onset muscle weakness.

## Case presentation

**Figure 1:**
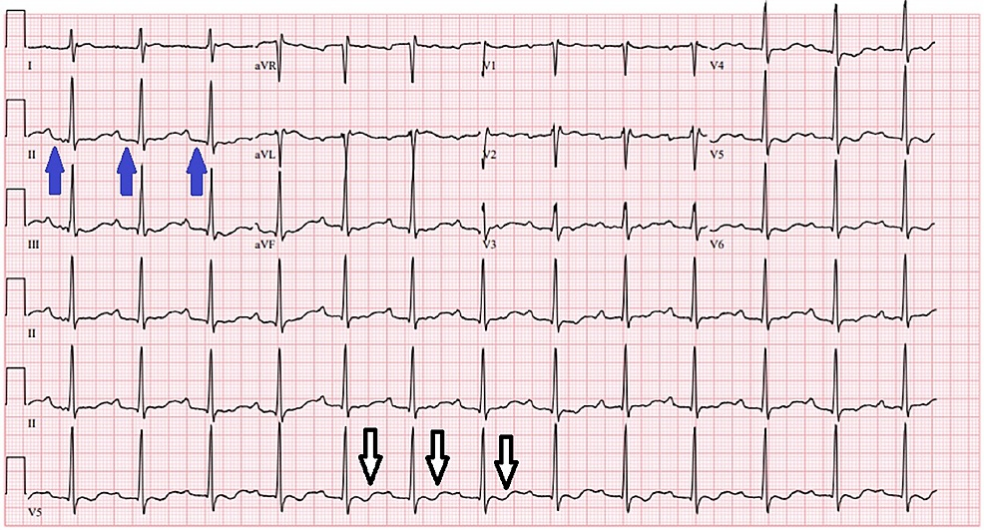
Electrocardiogram showing prolonged PR interval (blue arrows), and T-wave inversions (white arrows).

A 28-year-old athletic male with a non-significant past medical history presented with sudden onset generalized muscle paralysis four hours after a strenuous activity at the gym. He reported worsening severe weakness in his proximal muscles. He denied any prior episodes, seizures, or trauma. He also denied any smoking, drug, or supplement use. On presentation, vital signs were normal. He had decreased muscle power and tone in all extremities, more prominently in proximal muscles with hypoactive deep tendon reflexes. Laboratory workup was remarkable for severe hypokalemia 2 mmol/L, and hypomagnesemia 1.6 mg/dL. Urine potassium 16.4 mEq/mmol, urine creatinine 54.6 mg/dL, urine potassium with potassium-creatinine concentration ratio 0.3 mEq/mmol. Thyroid function was normal. An electrocardiogram showed prolonged PR interval, T-wave inversion, QT prolongation, and U waves (Figure 1). The patient received intravenous potassium replacement and was monitored for 24 hours until his potassium level normalized and all his symptoms resolved. Laboratory workup after treatment was consistent with electrolyte panel and EKG which all came back normal.

## Discussion

Focal muscle paralysis is an alarming symptom with a broad differential diagnosis including stroke, Todd’s paralysis, cataplexy, acute transverse myelitis, polymyositis, poliomyelitis, porphyria, and drug/alcohol intoxication. Although extremely rare, periodic paralysis disorders must be considered as a differential diagnosis for weakness so that proper treatment can be initiated quickly.

HPP is the most common of the periodic paralyses with a prevalence of 0.001% [3]. HPP is usually precipitated by vigorous exercise, fasting, or high-carbohydrate meals. This form of periodic paralysis is thought to be the result of disordered cellular potassium regulation secondary to sodium and calcium channelopathy in the setting of genetic mutations (CACNA1S and SCN4A) causing an abnormal potassium influx leading to hypokalemia and inefficient muscle contraction [4]. The clinical presentation ranges from mild fatigue and constipation to paralysis with cardiac arrhythmias.

HPP definitive diagnosis can be achieved through electromyography and muscle biopsy with provocative testing, however, potassium levels, thyroid profile, and electrocardiogram are the minimum indicated laboratory investigations. Acute treatment includes plasma potassium replacement, maintenance of acid-base balance, cardiac monitoring, and cautious use of neuromuscular blocking agents [5]. Preventative treatment against recurrent HPP has been successful with spironolactone and acetazolamide.

## Conclusions

In conclusion, HPP should be considered as a differential diagnosis of sudden onset paralysis as failure to properly diagnose and treat such a condition can be fatal. Raising awareness about HPP, especially in low-risk young patients without comorbidities can protect them from unnecessary radiation exposure.
